# Involvement of Prolactin-Releasing Peptide in the Activation of Oxytocin Neurones in Response to Food Intake

**DOI:** 10.1111/jne.12019

**Published:** 2013-04-12

**Authors:** M Yamashita, Y Takayanagi, M Yoshida, K Nishimori, M Kusama, T Onaka

**Affiliations:** * Division of Brain and Neurophysiology, Department of Physiology, Jichi Medical UniversityShimotsuke-shi, Japan; † Department of Dentistry, Oral and Maxillofacial Surgery, Jichi Medical UniversityShimotsuke-shi, Japan; ‡ Department of Molecular and Cell Biology, Graduate School of Agricultural Science, Tohoku UniversityMiyagi-ken, Japan

**Keywords:** oxytocin, PrRP, vasopressin, food intake, CCK

## Abstract

Food intake activates neurones expressing prolactin-releasing peptide (PrRP) in the medulla oblongata and oxytocin neurones in the hypothalamus. Both PrRP and oxytocin have been shown to have an anorexic action. In the present study, we investigated whether the activation of oxytocin neurones following food intake is mediated by PrRP. We first examined the expression of PrRP receptors (also known as GPR10) in rats. Immunoreactivity of PrRP receptors was observed in oxytocin neurones and in vasopressin neurones in the paraventricular and supraoptic nuclei of the hypothalamus and in the bed nucleus of the stria terminalis. Application of PrRP to isolated supraoptic nuclei facilitated the release of oxytocin and vasopressin. In mice, re-feeding increased the expression of Fos protein in oxytocin neurones of the hypothalamus and bed nucleus of the stria terminalis. The increased expression of Fos protein in oxytocin neurones following re-feeding or i.p. administration of cholecystokinin octapeptide (CCK), a peripheral satiety factor, was impaired in PrRP-deficient mice. CCK-induced oxytocin increase in plasma was also impaired in PrRP-deficient mice. Furthermore, oxytocin receptor-deficient mice showed an increased meal size, as reported in PrRP-deficient mice and in CCK_A_ receptor-deficient mice. These findings suggest that PrRP mediates, at least in part, the activation of oxytocin neurones in response to food intake, and that the CCK–PrRP–oxytocin pathway plays an important role in the control of the termination of each meal.

Oxytocin is synthesised in magnocellular neurones of the supraoptic nucleus (SON) and in magnocellular and parvocellular neurones of the paraventricular nucleus (PVN) in the hypothalamus. Small amounts of oxytocin are also generated in the bed nucleus of the stria terminalis (BNST). Oxytocin in magnocellular neurones is transported to the posterior lobe of the pituitary gland and released into peripheral blood following a variety of physiological stimuli, such as suckling and stretching of the uterine cervix 1. Oxytocin is also released within various brain regions from axonal terminals of oxytocin neurones and from dendrites or cell bodies of magnocellular oxytocin neurones of the hypothalamus 2, and it has been implicated in various functions 1, including social behaviour 3–5, anxiety and stress 6,7, as well as pain and energy metabolism 7,8.

Oxytocin has been shown to modulate food intake 8. The central administration of oxytocin reduces food intake 9,10. Food intake or administration of cholecystokinin octapeptide (CCK), which is a peripheral satiety factor released from the gut following food intake, activates oxytocin neurones in the hypothalamus 11–14, whereas fasting reduces the amounts of oxytocin mRNA in the hypothalamus 15.

Prolactin-releasing peptide (PrRP) has also been implicated in the control of food intake 16. Administration of PrRP induces anorexia 17, and PrRP- or PrRP receptor-deficient mice 18–20 show hyperphagia and obesity. Food intake 18 or CCK administration 21 activates PrRP-synthesising neurones in the nucleus tractus solitarii (NTS), whereas fasting reduces the amount of PrRP mRNA in the NTS 17. Some of the PrRP-synthesising neurones project to magnocellular oxytocin neurones in the SON 22 and to neurones in the PVN 23. The administration of PrRP facilitates oxytocin release into peripheral blood 24. It is thus possible that food intake activates PrRP neurones in the NTS and then stimulates hypothalamic oxytocin neurones, resulting in the termination of food intake.

To test the hypothesis that food intake activates oxytocin neurones via PrRP neurones, we first examined whether oxytocin or vasopressin neurones express PrRP receptors (also known as GPR10) and whether PrRP application facilitates release of oxytocin and vasopressin from hypothalamic explants in rats. We then examined the expression of Fos protein, a marker of neural activation, in oxytocin neurones of PrRP-deficient mice following food intake or administration of CCK. The pattern of food intake in oxytocin receptor-deficient mice was also investigated.

## Materials and methods

### Animals

Rats and mice were housed under a 12 : 12 h light/dark cycle (lights on 07.30 h) at 22 ± 2 °C and 40–70% relative humidity. Food and water were available *ad lib*., unless specified otherwise. Animal experiments were carried out after receiving approval from the Institutional Animal Experiment Committee of Jichi Medical University and were in accordance with the Institutional Regulations for Animal Experiments and Fundamental Guidelines for Proper Conduct of Animal Experiments and Related Activities in Academic Research Institutions under the jurisdiction of the Ministry of Education, Culture, Sports, Science and Technology.

### Immunocytochemical detection of PrRP receptor in oxytocin-immunoreactive (-IR) or vasopressin-IR neurones

For immunochemical detection of PrRP receptor in oxytocin-IR or vasopressin-IR neurones, male rats (11 weeks old, SLC: Wistar, Japan SLC, Shizuoka, Japan) were anaesthetised with pentobarbital (50 mg/kg body weight; Nembutal; Dainippon Sumitomo Pharma Co., Ltd., Osaka, Japan) and perfused transcardially with heparinised saline (20 U/ml) followed by 4% paraformaldehyde in 0.1 m phosphate buffer for 15 min. Brains were removed from the skulls, post-fixed in 4% paraformaldehyde overnight, then placed in 30% sucrose in 0.1 m phosphate buffer until they sank, and frozen in dry ice. The BNST and hypothalamic part of each frozen brain were sectioned coronally at 30 μm and processed for immunochemical detection of PrRP receptor and oxytocin or vasopressin. Sections were first incubated with rabbit anti-PrRP receptor antibody (dilution 1: 200) for 2 days at 4 °C followed by incubation with Alexa Fluor® 488 goat anti-rabbit immunoglobulin (Ig)G (dilution 1: 500, Life Technologies Japan Ltd, Tokyo, Japan) for 2 h at room temperature. Anti-PrRP receptor antibody was generated against a peptide of an predicted extracellular domain sequence of rat PrRP receptor, NH_2_-KPHDVRLCEEFWGSQERQRQ-COOH. For detection of oxytocin or vasopressin, sections were incubated with mouse anti-oxytocin antibody (dilution 1: 1000; Millipore, Tokyo, Japan) or guinea pig anti-vasopressin antibody (dilution of 1: 5000, Peninsula Laboratories, San Carlos, CA, USA) for 2 days at 4 °C and then with Alexa Fluor® 546 donkey anti-mouse IgG (dilution 1: 500; Life Technologies Japan Ltd) or Alexa Fluor® 568 goat anti-guinea pig IgG (dilution 1: 500, Life Technologies Japan Ltd) for 2 h at room temperature. For pre-absorption control study, the PrRP receptor antibody solution was pre-incubated with the antigen (84.29 μg/ml) before immunocytochemical procedures. Sections were observed using a confocal microscope (TCS SP5; Leica Microsystems, Wetzlar, Germany). Percentages of oxytocin and vasopressin neurones showing immunoreactivity for PrRP receptors were calculated. The number of animals was four or five.

### Peptide release from isolated SONs

Brains were obtained from male rats (SLC: Wistar, Japan SLC) at 5–6 weeks of age by decapitation. Two blocks of basal hypothalamic tissues (2 × 1 × 1 mm) containing the SONs were dissected out from each brain under a dissecting microscope 25. The blocks were transferred to normal Locke buffer solution (140 mm NaCl, 5 mm KCl, 1.2 mm MgCl_2,_ 1.8 mm CaCl_2,_ 10 mm glucose, 10 mm HEPES, pH 7.25 with Tris) and maintained at 37 °C throughout the experiments. Normal Locke buffer was changed at 5-min intervals for 90 min before collecting the samples. After the pre-incubation period, samples were collected every 5 min in a volume of 200 μl until the end of the experiment. PrRP at a concentration of 0.1, 1 or 10 μm was applied for 15 min. The collected samples were immediately stored at −80 °C until radioimmunoassay for oxytocin and vasopressin. Stimulus-evoked hormone release during drug application at each dosage is presented as the amount of hormone released compared to basal release (fraction before application of PrRP or the vehicle). Total sums of differences in oxytocin and vasopressin contents of the five consecutive samples (25-min samples) following the initiation of PrRP application compared to the samples before PrRP were calculated. The number of animals per group was eight.

### Measurement of oxytocin and vasopressin content

Amounts of oxytocin and vasopressin were determined by radioimmunoassays with specific anti-oxytocin (rabbit polyclonal antiserum kindly provided by Professor T. Higuchi, University of Fukui, Fukui, Japan) and anti-vasopressin antibodies (Mitsubishi-Yuka Co., Tokyo, Japan) as described previously 26,27. Coefficients of intra- and inter-assay variations were 4% and 10% for oxytocin, and 6% and 14% for vasopressin, respectively. The minimum detection limits were 2 pg/ml for oxytocin and 0.5 pg/ml for vasopressin. PrRP at a concentration of 10 μm showed no cross-reactions in the present radioimmunoassays.

### Immunocytochemical detection of Fos protein in oxytocin-IR or vasopressin-IR neurones following food intake

Male mice (10 weeks old, C57BL/6N mice; Charles River Laboratories, Hino, Japan) were individually housed and fasted for 24 h. Experimental groups of mice were re-fed from 10.00 h until 12.00 h or from 19.30 h until 21.30 h for 120 min. Control animals were kept in their individual home cages until brain sampling (12.00 or 21.30 h). The number of mice in each group was eight.

In another series of experiments, PrRP-deficient male mice 18 or their littermate wild-type male mice (11 weeks old) were fasted for 24 h. Experimental groups of mice were re-fed for 120 min (19.30–21.30 h, n = 5–8 in each group).

Two hours after the initiation of re-feeding or at the corresponding time (12.00 or 21.30 h), experimental or control mice were anaesthetised with Avertin (200 mg/kg body weight, i.p.; tribromoethanol; Sigma-Aldrich, St Louis, MO, USA) and perfused transcardially with heparinised saline (20 U/ml) followed by 4% paraformaldehyde in 0.1 m phosphate buffer for 15 min. Brains were removed from the skulls, post-fixed in 4% paraformaldehyde overnight, then placed in 30% sucrose in 0.1 m phosphate buffer until they sank, and frozen in dry ice. The hypothalamic part of each frozen brain was sectioned coronally at 30 μm and processed for immunochemical detection of Fos protein, oxytocin, or vasopressin, as described previously 28. In brief, sections were incubated with a rabbit polyclonal antibody raised against the N-terminal 4–17 Fos peptide sequence (Ab-5; Oncogene Science; Cambridge, MA, USA) diluted at 1: 10 000 for 2 days at 4 °C. Immunoreactivity was visualised by sequential overnight incubation with peroxidase-labelled goat anti-rabbit IgG (dilution 1: 500; Vector Laboratories, Peterborough, UK) at 4 °C and 3,3′-diaminobenzidine tetrahydrochloride (DAB) with nickel sulphate. The sections were then processed for detection of oxytocin using an antibody against oxytocin (dilution 1: 5000; Chemicon International Inc., Temecula, CA, USA,) or for detection of vasopressin using anti-vasopressin antiserum (dilution 1: 40 000; Chemicon International Inc.). After 48 h of incubation at 4 °C with the first antibodies, sections were incubated with biotinylated anti-rabbit IgG (dilution 1: 500; Vector Laboratories) for 2 h at room temperature and with avidin biotinylated horseradish peroxidase complex (dilution 1: 50; Vector Laboratories) for 30 min. Oxytocin or vasopressin immunoreactivity was visualised as a brown cytoplasmic precipitate with DAB. Eight sections were examined for the hypothalamic PVN or SON at an interval of 90 μm in each mouse. For the BNST, two sections at an interval of 90 μm were examined per mouse.

### CCK administration

PrRP-deficient male mice or their littermate wild-type male mice (18 weeks old) were intraperitoneally administered CCK (Peptide Institute, Minoh, Japan) at a dose of 20 μg/kg or a vehicle (0.9% NaCl). Ten minutes after the injection, trunk blood was collected by decapitation for measuring plasma concentration of oxytocin. The number of animals per group was four to seven.

In another series of experiments, PrRP-deficient mice or their littermate wild-type mice (25–31 weeks old) were i.p. injected with CCK (20 μg/kg) or the vehicle. Two hours after the injection, animals were perfused transcardially with 4% paraformaldehyde under anaesthesia. Brains were processed for immunocytochemical detection of Fos protein and oxytocin in the hypothalamus.

### Measurements of meal size and meal frequency

Food intake of oxytocin receptor-deficient male mice 29 or their littermate wild-type male mice (18 weeks old) was recorded every minute by an automatic food counter (O'Hara and Co., Ltd., Tokyo, Japan) 18. Meal initiation was defined as more than 50 mg pellets within 10 min. Once a meal was initiated, meal termination was defined as the onset of a 10-min interval with no intake. The number of animals per group was six (wild-type mice) or ten (oxytocin receptor-deficient mice).

### Statistical analysis

Data are expressed as the mean ± SEM. The time course data of experiments with isolated SONs were analysed with one-factor repeated measures anova followed by Dunnett's t-test. Data for hormone release from isolated SONs with various dosages of PrRP were analysed by one-factor anova followed by Dunnett's t-test. Other data were analysed using one-factor anova followed by Fisher's protected least significant difference in the case of multiple comparison or Student's t-test in the case of a comparison of two groups. P < 0.05 was considered statistically significant.

## Results

### Expression of PrRP receptors in the hypothalamic PVN, SON and BNST of rats

**Fig. 1:**
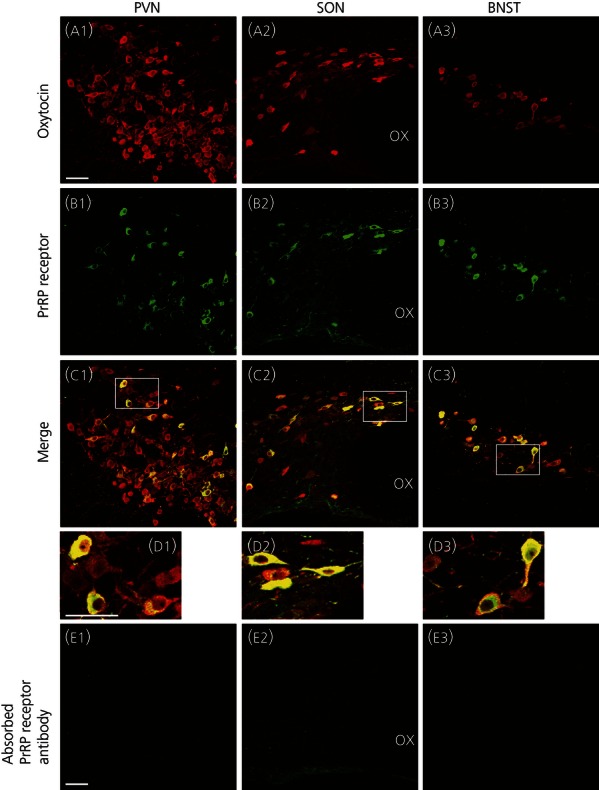
Expression of prolactin-releasing peptide (PrRP) receptors in oxytocin-immunoreactive (-IR) neurones in the hypothalamus or bed nucleus of the stria terminalis (BNST) in rats. Immunoreactivity for oxytocin (red fluorescence, a) or PrRP receptors (green fluorescence, b) is shown. Double-labelled cells are shown in yellow (c, d). Areas enclosed by white rectangles in the merged images (c) are shown at a higher magnification (d). Immunoreactive PrRP receptors were observed in oxytocin-IR cells of the paraventricular nucleus (PVN, a1, b1, c1, d1), supraoptic nucleus (SON, a2, b2, c2, d2) and BNST (a3, b3, c3, d3) in rats. All labellings for PrRP receptors were prevented by pre-absorption of anti-PrRP receptor antibody with an excess of the synthetic antigen peptide (e). The third cereberoventricle (not shown) is located on the right side of each picture. OX, optic chiasma. Magnifications in (a–c) and (e) are equivalent. Scale bars = 50 μm.

**Fig. 2:**
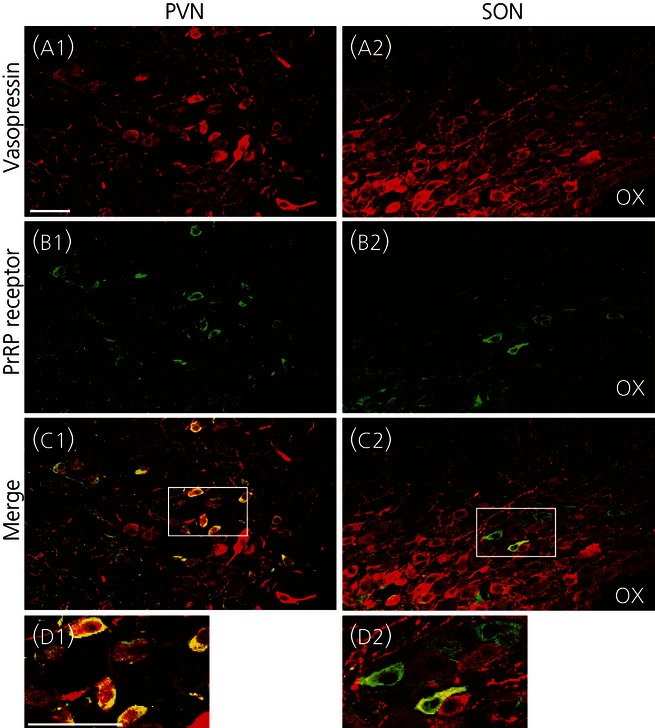
Expression of prolactin-releasing peptide (PrRP) receptors in vasopressin-immunoreactive (-IR) neurones in the hypothalamus of rats. Immunoreactivity for vasopressin (red fluorescence, a) or PrRP receptors (green fluorescence, b) is shown. Double-labelled cells are shown in yellow (c, d). Areas enclosed by white rectangles in the merged images (c) are shown at a higher magification (d). Immunoreactive PrRP receptors were observed in vasopressin-IR cells of the PVN and SON. OX, optic chiasma. Scale bars = 50 μm.

To determine whether PrRP receptors are expressed in oxytocin-IR or vasopressin-IR neurones, sections containing the hypothalamus and BNST were processed for double immunocytochemical detection of oxytocin and PrRP receptors. Immunoreactivity of PrRP receptors was observed in the PVN, SON and BNST (Fig. 1). In the PVN, SON or BNST, a considerable number of oxytocin-IR neurones and a smaller number of vasopressin-IR neurones showed immunoreactivity for PrRP receptors (Figs 1 and 2). The percentage of oxytocin neurones showing immunoreactivity for PrRP receptors was 46 ± 5%, 31 ± 4% and 54 ± 5% in the PVN, SON and BNST, respectively. The percentage of vasopressin neurones showing immunoreactivity for PrRP receptors was 19 ± 4% and 7 ± 5% in the PVN and SON, respectively. Oxytocin-IR cells showing PrRP receptor immunoreactivity were found both in the magnocellular and parvocellular regions of the PVN. All labellings for PrRP receptors were prevented by pre-absorption of anti-PrRP receptor antibody with an excess of the synthetic antigen peptide (Fig. 1e), whereas oxytocin labellings with anti-oxytocin antibody were not disturbed (data not shown).

### Oxytocin and vasopressin release from isolated SONs of rats

**Fig. 3:**
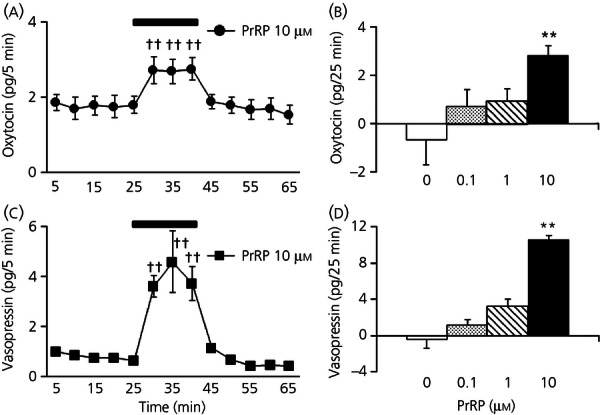
Oxytocin or vasopressin release from isolated supraoptic nuclei (SONs) of rats by application of prolactin-releasing peptide (PrRP). Hormonal concentrations in the perfusates of isolated SONs of rats before or following PrRP application (10 μm) (a, c) and total amounts of hormones released in response to PrRP application (0, 0.1, 1 or 10 μm) (b, d) are shown. PrRP was applied during the time period between 25 min and 40 min. Total sum of hormone increases in perfusates collected between 30 and 50 min (5 samples) compared to the sample collected before PrRP application (at the time of 25 min) is shown (b, d). Following application of PrRP to the isolated SON, oxytocin or vasopressin concentrations in the perfusates of the explants were significantly increased in a dose-related fashion. ^††^P < 0.01 compared to data obtained before PrRP application (at the time point of 25 min). **P < 0.01 compared to vehicle-treated control (n = 8).

We then examined whether application of PrRP facilitates oxytocin or vasopressin release from isolated SON explants of rats. Following application of PrRP to isolated SONs, oxytocin or vasopressin concentrations in the perfusates of the explants were significantly increased in a dose-related fashion (Fig. 3). These results suggest that PrRP facilitates somato-dendritic release of oxytocin and vasopressin within the hypothalamus.

### Expression of Fos protein in oxytocin-IR neurones following food intake in mice

We then investigated whether food intake activates oxytocin-containing neurones by examining expression of Fos protein in oxytocin-IR cells following re-feeding in mice.

**Fig. 4:**
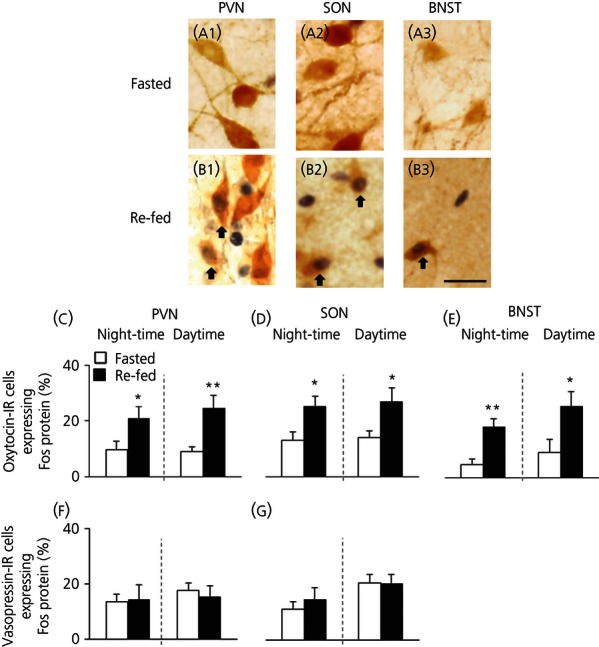
Expression of Fos protein in oxytocin-immunoreactive (-IR) or vasopressin-IR neurones of the hypothalamus or bed nucleus of the stria terminalis (BNST) following re-feeding in mice. Images of oxytocin-IR cells and Fos protein immunoreactivity in the paraventricular nucleus (PVN) (a1, b1), supraoptic nucleus (SON) (a2, b2) and BNST (a3, b3) of night-time-fasted or re-fed mice and the percentages of oxytocin-IR or vasopressin-IR cells expressing Fos protein (c–g) are shown. Mice were fasted for 24 h and re-fed for 2 h in the daytime or night-time. The percentage of oxytocin-IR neurones expressing Fos protein was significantly increased in the PVN, SON or BNST after re-feeding in the daytime or at night-time. There were no significant differences in the percentages of Fos-positive vasopressin-IR neurones. Brown cytoplasmic reactions indicate oxytocin immunoreactivity and dark nuclear reactions indicate Fos immunoreactivity. Double-labelled neurones are indicated by arrows (b). Scale bar = 20 μm *P < 0.05 and **P < 0.01 compared to fasted mice (n = 8).

The percentage of oxytocin neurones expressing Fos protein in the hypothalamic PVN, SON or BNST was significantly increased following re-feeding for 2 h during the night-time or daytime (night-time; P = 0.02 for PVN, P = 0.01 for SON, P = 0.001 for BNST, daytime; P = 0.005 for PVN, P = 0.03 for SON, P = 0.03 for BNST) (Fig. 4). By contrast, there was no significant difference in the percentage of vasopressin-IR cells expressing Fos protein in the hypothalamus. The total amount of food intake during the 2-h period of re-feeding at the beginning of night-time was 0.81 ± 0.15 g and that following re-feeding in the daytime was 0.49 ± 0.04 g. These results suggest that food intake activates oxytocin neurones in the hypothalamus and BNST.

### Impaired activation of oxytocin-IR neurones following food intake in PrRP-deficient mice

We then examined whether activation of oxytocin-IR neurones in response to food intake was impaired in PrRP-deficient mice.

**Fig. 5:**
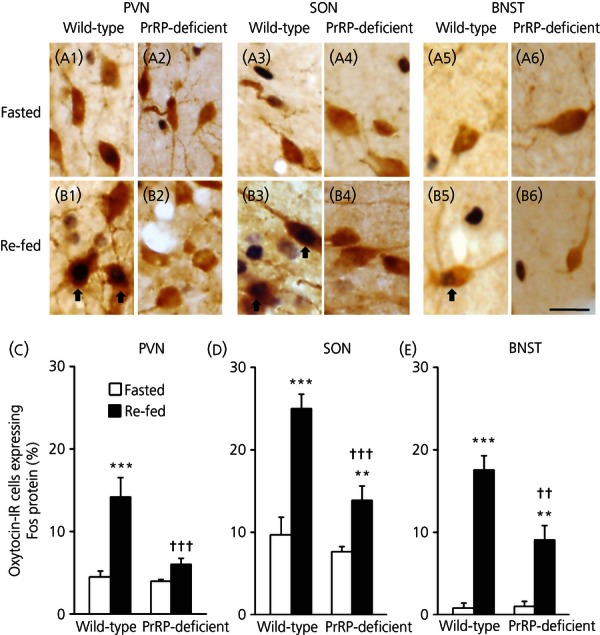
Expression of Fos protein in oxytocin-immunoreactive (-IR) neurones of the hypothalamus or bed nucleus of the stria terminalis (BNST) following re-feeding in prolactin-releasing peptide (PrRP)-deficient mice. Images of oxytocin-IR cells and Fos protein immunoreactivity in the paraventricular nucleus (PVN) (a1, a2, b1, b2), supraoptic nucleus (SON) (a3, a4, b3, b4) and BNST (a5, a6, b5, b6) of wild-type or PrRP-deficient mice in fasted or re-fed groups and the percentages of oxytocin-IR cells expressing Fos protein (c–e) are shown. The percentage of oxytocin-IR neurones expressing Fos protein following re-feeding was significantly decreased in the PVN, SON or BNST of PrRP-deficient mice compared to that in wild-type animals. Scale bar = 20 μm **P < 0.01 and ***P < 0.001 compared to fasted mice. ^††^P < 0.01 and ^†††^P < 0.001 compared to corresponding groups of wild-type mice (n = 5–8). Arrows indicate double-labelled neurones.

In PrRP-deficient mice, the percentages of oxytocin-IR neurones expressing Fos protein in the PVN, SON and BNST following re-feeding were significantly lower than those in the wild-type animals (P = 0.0002 for PVN, P = 0.0001 for SON, P = 0.007 for BNST) (Fig. 5). The total number of oxytocin-IR neurones was not significantly different among wild-type and PrRP-deficient animals of the control and re-fed groups. These results suggest that PrRP is essential for full activation of oxytocin neurones in response to food intake.

### Impaired activation of oxytocin-IR neurones and facilitation of oxytocin release in response to CCK in PrRP-deficient mice

Food intake induces release of CCK, and administration of CCK activates PrRP-expressing neurones in the medulla oblongata 21 and oxytocin neurones in the hypothalamus 11. We thus examined whether activation of oxytocin-IR neurones and facilitation of oxytocin release in response to CCK were impaired in PrRP-deficient mice.

**Fig. 6:**
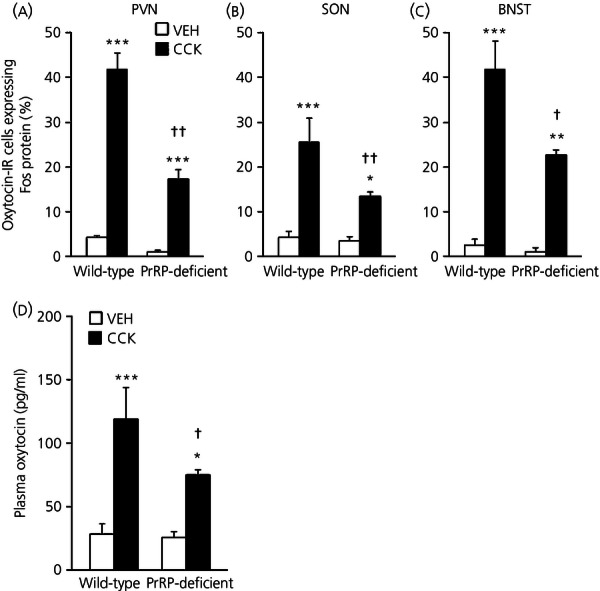
Expression of Fos protein in oxytocin-immunoreactive (-IR) neurones and plasma oxytocin concentrations following administration of cholecystokinin octapeptide (CCK) in prolactin-releasing peptide (PrRP)-deficient mice. The percentage of oxytocin neurones expressing Fos protein following i.p. CCK was significantly decreased in the paraventricular nucleus (PVN) (a), supraoptic nucleus (SON) (b) or bed nucleus of the stria terminalis (BNST) (c) of PrRP-deficient mice compared to that in wild-type animals. Plasma oxytocin concentrations following CCK administration were lower in PrRP-deficient mice than in wild-type animals (d). *P < 0.05, **P < 0.01 and ***P < 0.001 compared to vehicle (VEH)-injected mice. ^†^P < 0.05 and ^††^P < 0.01 compared to wild-type mice (n = 4–7).

The percentages of oxytocin-IR neurones expressing Fos protein in the PVN, SON and BNST were increased following the administration of CCK. The increase was significantly smaller in PrRP-deficient mice than in wild-type animals (P = 0.001 for PVN, P = 0.002 for SON, P = 0.01 for BNST) (Fig. 6).

Plasma oxytocin concentrations were significantly increased following CCK administration (Fig. 6), and the CCK-induced oxytocin increase was significantly reduced in PrRP-deficient mice (P = 0.02). These results suggest that PrRP is also essential for full activation of oxytocin neurones and oxytocin release in response to CCK.

### Meal pattern in oxytocin receptor-deficient mice

CCK plays an important role in determining the size of each meal 30. CCK_A_ receptor-deficient mice 31 and PrRP-deficient animals 18 show increased meal size. We thus examined the meal pattern in oxytocin receptor-deficient mice.

**Fig. 7:**
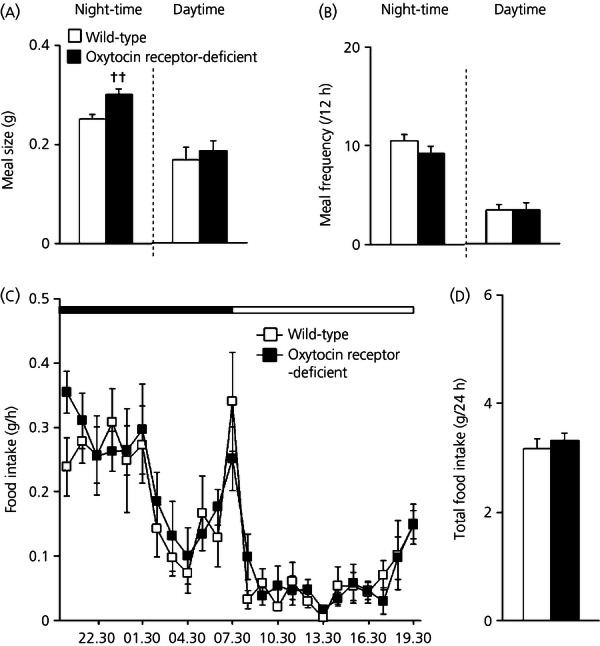
Meal size in oxytocin receptor-deficient mice. Meal size (a) but not meal frequency (b) during the night-time was increased in oxytocin receptor-deficient mice comapred to that in wild-type animals. There was no significant difference in the amount of food intake at any time of the day (c) or the total amount of food intake per day (d) between genotypes. ^††^P < 0.01 to compared with wild-type mice (n = 6 or 10).

Meal size during the night-time [P = 0.009, 0.3 ± 0.01 g (oxytocin receptor-deficient mice) versus 0.25 ± 0.01 g (wild-type mice)] or for a 24-h period [P = 0.018, 0.27 ± 0.01 g (oxytocin receptor-deficient mice) versus 0.23 ± 0.01 g (wild-type mice)] was significantly larger in oxytocin receptor-deficient mice than in wild-type animals, although the difference in meal size during the daytime was not statistically significant [0.19 ± 0.02 g (oxytocin receptor-deficient mice) versus 0.17 ± 0.03 (wild-type mice)] (Fig. 7). Meal frequency was not statistically different between oxytocin receptor-deficient and wild-type animals. There was no significant difference in amounts of food intake at any time of the day or in the total amounts of food intake per day between oxytocin receptor-deficient and wild-type mice. These results suggest that the oxytocin receptor as well as PrRP or CCK is essential for determining meal size.

## Discussion

In the present study, oxytocin-IR neurones expressed PrRP receptors and the application of PrRP facilitated oxytocin release from the hypothalamic explants. We previously showed that oxytocin neurones in the hypothalamus receive projections from PrRP neurones in the medulla oblongata 22 and that PrRP neurones are activated by food intake 18. Food intake also activated oxytocin-IR neurones in the hypothalamus, consistent with previously reported data 32,33. The present study further demonstrated that activation of oxytocin-IR neurones in response to food intake or CCK administration was impaired in PrRP-deficient mice. All of these findings suggest that food intake activates hypothalamic oxytocin neurones via medullary PrRP neurones. Furthermore, the results of the present study show that oxytocin receptor-deficient mice had increased amounts of meal size similar to the phenotypes of PrRP-deficient mice 18, suggesting that oxytocin receptors play an important role in the termination of each meal.

PrRP neurones are localised in A2 noradrenergic neurones of the NTS and in A1 noradrenergic neurones of the ventrolateral medulla. These noradrenergic neurones in either region project to the SON or PVN 12,34. In the present study, it was found that oxytocin neurones express PrRP receptors and that PrRP application facilitated oxytocin release from the SON. Most of the medullary PrRP neurones activated by food intake have been shown to be localised in the NTS 18, and PrRP deficiency blocked activation of oxytocin neurones following food intake. All of these findings suggest the involvement of PrRP neurones of the NTS in the activation of oxytocin neurones following food intake. By contrast, the role of PrRP neurones in the ventrolateral medulla remains to be clarified. Neurones in the ventrolateral medulla directly project to hypothalamic vasopressin neurones 35 and, in the present study, some vasopressin-IR neurones in the hypothalamus expressed PrRP receptors and PrRP application facilitated vasopressin release from the SON. Vasopressin released within the hypothalamus has been shown to modulate activity of vasopressin neurones 36. Consistent with the present data suggesting that PrRP activates SON vasopressin neurones, microinjections of PrRP into the PVN have been reported to facilitate vasopressin release into the peripheral circulation 23. Haemorrhage activates PrRP neurones in the ventrolateral medulla 37 and vasopressin neurones in the hypothalamus 23. It is interesting to speculate that PrRP neurones in the ventrolateral medulla might play a role in activation of vasopressin neurones following haemorrhage.

Meal size is regulated by satiety signals that terminate each meal. One important satiety signal is CCK 30, which is released from the gut after each meal and acts on CCK_A_ receptors on afferent fibres of the gastric vagus nerve projecting to the medulla oblongata. CCK activates PrRP neurones in the medulla oblongata 38 and oxytocin neurones in the hypothalamus 12. In the present study, activation of oxytocin-IR neurones and oxytocin release in response to CCK were impaired in PrRP-deficient mice, suggesting that CCK activates oxytocin neurones, at least in part, via PrRP neurones. Both CCK_A_ receptor-deficient mice 31 and PrRP-deficient mice 18 show an increase in meal size but not in meal frequency. A nonselective antagonist that blocks either oxytocin receptor or vasopressin V1 receptor 39 has been shown to increase meal size 40 and activation of vasopressin V1 receptors induces anorexia 41,42. The results of the present study showing that oxytocin receptor-deficient mice had an increased meal size clearly indicate the involvement of oxytocin receptors in determining meal size. All of these findings are consistent with the idea that the CCK–PrRP–oxytocin pathway is critical for controlling meal size. However, the findings do not exclude the possible involvement of other anorexic substances such as glucagon-like peptide-1 (GLP-1), α-melanocyte-stimulating hormone (α-MSH) derived from proopiomelanocortin (POMC), cocaine- and amphetamine-regulated transcript (CART), corticotophin-releasing hormone (CRH) and nesfatin in CCK-induced anorexia because CCK administration has been shown to activate neurones containing these peptides 43,44–46. Further study is necessary to clarify roles of these peptides.

The sites of the action of oxytocin for inducing anorexia remain unclear. However, the NTS has been proposed to be a site of the action of oxytocin. Parvocellular oxytocin neurones projecting to the NTS are activated following administration of an anorexic dose of leptin 47, and either application of a nonselective oxytocin antagonist into the fourth ventricle 47,48 or destruction of oxytocin-responsive cells in the NTS by the administration of cytotoxic saporin-conjugated oxytocin 48 increases food intake. Oxytocin administration activates GLP-1- or POMC-synthesising neurones in the medulla oblongata 49,50, and the anorexic actions of oxytocin are impaired by antagonists of GLP-1 receptors or MC3/4R 49,50. All of these findings suggest that the NTS projected by parvocellular oxytocin neurones of the hypothalamic PVN is a site of anorexic action of oxytocin. By contrast, the present study showed that food intake activated not only parvocellular oxytocin neurones in the PVN, but also magnocellular oxytocin neurones in the SON. Oxytocin is not only released from the axonal terminals in the posterior lobe of the pituitary, but also from the dendrites and cell bodies of magnocellular neurones in the hypothalamus. The results of the present study show that PrRP facilitated oxytocin release from the SON slice preparation that contains only somata and dendrites but not axon terminals of oxytocin neurones, suggesting that PrRP induces somato-dendritic oxytocin release within the hypothalamus. Oxytocin receptors are localised within the hypothalamus, including the ventromedial hypothalamus and arcuate nucleus 51, which control food intake. The potential role of somato-dendritic oxytocin release during food intake remains to be determined.

In the hypothalamic preparations of the present study, PrRP at concentrations of 0.1–10 μm was used to induce the release of oxytocin and vasopressin from SON preparations. The concentrations of 1–10 μm are within the range estimated to reach the brain after i.c.v. injections of 1–10 nmol of PrRP, which have been reported to induce anorexia and neuroendocrine responses 18,21,24,52. However, PrRP receptors have nanomolar affinity for PrRP. PrRP concentrations at sites where PrRP acts are unknown. Further studies are necessary to clarify the precise roles of PrRP receptors and the physiological significance of dendritic release of oxytocin or vasopressin following PrRP administration.

In the present study, food intake and CCK administration activated oxytocin-IR neurones in the BNST, consistent with the results of previous studies showing that palatable food 53 or CCK 54 induces the expression of Fos protein in the BNST. BNST neurones receive projections from A2 noradrenergic neurones in the NTS and from A1 neurones in the ventrolateral medulla. Some of these noradrenergic neurones express PrRP and PrRP-IR fibres are found in the BNST 55. Food intake and CCK administration activate PrRP neurones in the medulla oblongata 18,21. The results of the present study showing that activation of oxytocin neurones in the BNST was impaired in PrRP-deficient animals suggest the involvement of PrRP projections from the medulla oblongata in the activation of oxytocin neurones in the BNST, although the role of oxytocin neurones in the BNST remains to be clarified. The BNST and noradrenergic projections to the BNST have been shown to be involved in the control of stress responses 56,57. Food intake 58 and oxytocin 6 affect stress responses. It is thus interesting to speculate that oxytocin neurones activated during food intake may play a role in modulation of stress responses by food intake. It is also possible that oxytocin neurones in the BNST affect food intake itself because the BNST has been implicated in food or reward seeking behaviour 59.

The present study suggests that PrRP has an important role in activation of oxytocin neurones following food intake. However, the data do not exclude other possible pathways to activate oxytocin neurones. In PrRP-deficient animals, activation of oxytocin neurones following food intake or CCK administration was reduced but was still observed, suggesting that additional factors other than PrRP are involved in activation of oxytocin neurones. Noradrenergic neurones in the medulla oblongata that project to the hypothalamus are activated following CCK administration, and CCK-induced activation of oxytocin neurones is impaired by an α1-adrenoceptor antagonist or local lesions of adrenergic projections to the hypothalamus 7,11,12. The data suggest the possible involvement of adrenergic transmission in the activation of oxytocin neurones following CCK administration. It is also possible that GLP-1, α-MSH, CART, CRH and nesfatin play a role. Food intake or CCK administration activates GLP-1-containing neurones in the NTS 43, POMC neurones in the arcuate nucleus or NTS 44, and hypothalamic neurones containing CART 45, CRH 46 or nesfatin 46,60. The administration of these peptides facilitates oxytocin release or activates oxytocin neurones in the hypothalamus 36,50,61,62. The roles of these anorexic peptides in activation of oxytocin neurones observed following CCK administration remain to be investigated. Oxytocin neurones may receive information on metabolic status of the body. The expression of oxytocin is regulated by a metabolic regulator, peroxisome proliferator-activated receptor coactivator-1a (PGC-1α, also known as PPARγC1α) 63. Hypothalamic injections of leucine, a physiological signal of hypothalamic amino acid availability, activate PVN oxytocin neurones 40. It is thus also possible that metabolic states might have contributed to the activation of oxytocin neurones observed following food intake. Plasma osmolality has been reported to increase following food intake, and a possible increase in osmolality has been proposed to be a cause of activation of the SON and PVN following food intake 33,64. However, it is unlikely that increased osmolality is the cause of activation of oxytocin neurones in the present conditions because vasopressin neurones were not activated and an increase in plasma osmolality should have activated vasopressin neurones, as well as oxytocin neurones.

By using PrRP-deficient mice and oxytocin receptor-deficient mice, the present study demonstrates that PrRP, at least in part, mediates the activation of oxytocin neurones in response to food intake and that the oxytocin receptor is essential for determining meal size. CCK_A_ receptor-deficient mice and rats 31,65, PrRP-deficient mice 18 and oxytocin receptor-deficient mice 66 (Fig. 7) all show an increased meal size and late-onset obesity, suggesting the importance of the CCK–PrRP–oxytocin pathway in the control of food intake, especially in the termination of each meal.
